# An *α*-fetoprotein-derived peptide reduces the uterine hyperplasia and increases the antitumour effect of tamoxifen

**DOI:** 10.1038/sj.bjc.6603882

**Published:** 2007-07-17

**Authors:** T T Andersen, J Georgekutty, L A DeFreest, G Amaratunga, A Narendran, N Lemanski, H I Jacobson, J A Bennett

**Affiliations:** 1 Center for Cardiovascular Sciences, Albany Medical College, Albany, NY 12208, USA; 2 Center for Immunology and Microbial Diseases, Albany Medical College, Albany, NY 12208, USA; 3 Department of Obstetrics, Gynecology and Reproductive Sciences, Albany Medical College, Albany, NY 12208, USA

**Keywords:** tamoxifen, breast cancer, α-fetoprotein, chemoprevention

## Abstract

Tamoxifen (Tam) is effective for the treatment and prevention of breast cancer. However, it has toxic drawbacks and has limited-duration utility because, over time, human tumours become refractory to Tam. Recently, a new nontoxic peptide, *α*-fetoprotein-derived peptide (AFPep) has been proposed for the treatment and prevention of breast cancer. The purpose of this paper is to determine whether combining AFPep with Tam would increase efficacy and reduce toxicity in experimental models of breast cancer. Low doses of AFPep and Tam were more effective in combination than either agent alone against breast cancer growth in cell culture, in tumour-xenografted mice, and in carcinogen-exposed rats. *α*-Fetoprotein-derived peptide interfered with Tam-induced uterine hyperplasia in immature mice, and showed no toxic effects. Unlike Tam, AFPep did not inhibit binding of oestradiol (E_2_) to oestrogen receptor (ER). Thus, these two agents utilise different mechanisms to interfere with ER functionality, yet work cooperatively to reduce breast cancer growth and alleviate Tam's troubling toxicity of uterine hyperplasia and appear to be a rational combination for the treatment of ER-positive breast cancer.

A peptide (*α*-fetoprotein-derived peptide (AFPep), sequence *cyclo*(EKTOVNOGN), where O is hydroxyproline) derived from the active site of *α*-fetoprotein (AFP) has been under investigation as a potential agent for prevention or therapy of oestrogen receptor-positive (ER+) breast cancer (Mesfin *et al*, 2000, 2001; Bennett *et al*, 2002, 2006; DeFreest *et al*, 2004). After identifying the active site of AFP as an eight-amino acid peptide (Mesfin *et al*, 2000), Mesfin went on to develop a more stable, cyclised peptide with substantial potential as a pharmaceutical agent (Mesfin *et al*, 2001). *α*-Fetoprotein-derived peptide has been shown to inhibit the growth of human breast cancer xenografts in mice (Mesfin *et al*, 2001), and prevent the development of carcinogen-induced mammary cancers in rats (Parikh *et al*, 2005). It was also shown to inhibit the growth of breast cancer that had become resistant to the cytostatic effects of tamoxifen (Tam; Bennett *et al*, 2002). *α*-Fetoprotein-derived peptide is active after oral administration (Bennett *et al*, 2002), and has not exhibited toxicity in any study to date.

Tamoxifen has been the most widely used and effective drug for the treatment of ER+ breast cancer for many years (Jordan, 1999a). It has been shown to inhibit breast cancer growth (Lippman and Bolan, 1975), inhibit breast cancer recurrences (Early Breast Cancer Trialists' Collaborative Group, 1992), and decrease the risk of primary breast cancers in high-risk patients (Fisher *et al*, 1998). However, as mentioned above, some ER+ breast cancers acquire resistance to Tam, and some are actually resistant to Tam before treatment (Jensen and DeSombre, 1996). Moreover, although Tam is relatively well tolerated, it is not without unwanted sequelae in some patients. These toxicities and side effects are generally dose-dependent and include uterine hyperplasia, which can progress to uterine cancer in a small percentage of patients; thrombo-embolic episodes that can progress to deep vein thrombosis, pulmonary embolism, and stroke in a small percentage of patients; and nonlife-threatening side effects such as hot flashes, fluid retention, and vaginal discharge (Mosby, 2005). Reducing Tam-induced toxicity and providing alternatives to Tam for Tam-resistant tumours would advance the treatment of breast cancer. We have been investigating AFPep for these purposes (Mesfin *et al*, 2000, 2001; Bennett *et al*, 2002, 2006; DeFreest *et al*, 2004; Parikh *et al*, 2005). However, as part of this investigation, it seemed reasonable to evaluate AFPep in combination with Tam in that it might add to the therapeutic activity of Tam, and perhaps might even reduce the toxicity or side effects of Tam. This report describes results of AFPep in combination with Tam in models of breast cancer therapy, prevention, and host toxicity.

## MATERIALS AND METHODS

### Materials

Carcinogen (*N*-methyl-*N*-nitrosourea, MNU) was obtained from the National Cancer Institute carcinogen repository (MRI Inc., Kansas City, MO, USA) and was dissolved in sterile physiological saline (1%, w/v), buffered to pH 5.0 with 3% acetic acid. Female Sprague–Dawley rats were obtained from Taconic Farms (Germantown, NY, USA) at 34 days of age and were placed immediately on a controlled diet (Agway Pro-Lab 2000; Agway Corporation, Syracuse, NY, USA), allowed free access to food and water, and maintained on a 12-h light–dark cycle at a constant temperature (22°C) for the duration of the study. Severe combined immune-deficient (SCID) mice and Swiss Webster mice were obtained from Taconic Farms and were maintained in individually ventilated cages. Cages, bedding, food, and water for mice were autoclaved. Mice were handled using sterile technique in a laminar flow biosafety cabinet. All animal protocols were approved by the Institutional Animal Care and Use Committee (IACUC) at Albany Medical College who are guided by the United States Public Health Service regulations on the humane care and use of laboratory animals, and these guidelines meet the standards required by the UKCCCR (Workman *et al*, 1998).

### Peptide synthesis

The AFP-derived peptide, *cyclo*(EKTOVNOGN), where O is hydroxyproline, was generated using FMOC solid-phase peptide synthesis employing the head-to-tail cyclisation method (Kates *et al*, 1993). After synthesis, the resin was washed with propanol and partially dried, and peptides were cleaved from the solid support and deprotected simultaneously with 10 ml of trifluoroacetic acid/thioanisole/anisole/ethanedithiol (90 : 5 : 2 : 3) per 0.5 g of resin for 5 h. Peptide was recovered from the liquid phase after repeated extraction, first with ether and then with ethyl acetate/ether (1.5 : 1). The peptide was dissolved in water, purified by reverse-phase HPLC, and then lyophilised. Biologically active AFPep can also be purchased from PolyPeptide Laboratories (Torrance, CA, USA) after synthesis by the tBOC method, or from Advanced ChemTech (Louisville, KY, USA) after synthesis by the FMOC method.

### Cell culture assay

The MCF-7 cells were maintained in monolayer culture in Dulbecco's modification of Eagle's medium (DMEM) supplemented with 5% fetal bovine serum, glutamine (2 mM), nonessential amino acids (1%), and bovine insulin (1 *μ*g ml^−1^). The T47D human breast cancer cells were maintained in monolayer culture in RPMI-1640 medium supplemented with 10% fetal bovine serum, 100 IU ml^−1^ penicillin, 100 *μ*g ml^−1^ streptomycin, 0.25 *μ*g ml^−1^ amphotericin B, and 8 *μ*g ml^−1^ bovine insulin in T-75 flasks with 2–3 medium changes per week. Cells were maintained at 37°C in an atmosphere of 5% CO_2_, 95% air. To evaluate the oestrogen-stimulated growth of these cells, they were released from monolayer using 0.25% trypsin/0.53 mM EDTA and suspended in oestrogen-free medium comprised of DMEM (high glucose, phenol red-free), supplemented with 10% charcoal-stripped bovine calf serum, 2 mM glutamine, 100 IU ml^−1^ penicillin, and 100 *μ*g ml^−1^ streptomycin. One millilitre containing 1.2 × 10^5^ cells was seeded into each well of a 24-well plate coated with collagen IV (BD Biosciences, Bedford, MA, USA). Beginning from 1 day after seeding, cells were treated daily for 7 days with AFPep (10^−6^ M), Tam (10^−8^ M), or the combination of AFPep plus Tam in the presence or absence of E_2_(10^−9^ M). In these groups exposed to E_2_, E_2_ at 10^−9^ M was added 1 h after each addition of AFPep and Tam. Control wells received the vehicle. Medium was changed every other day before treatment. One day after the last treatment, cells were trypsinised and resuspended in oestrogen-free medium, and counted using a haemacytometer following dilution with trypan blue. Viable cell number is reported.

### Binding to oestrogen receptor

Rabbit uteri (Pel-Freez Biological, Rogers, AR, USA) were used as a source of ER. Uteri were pulverised in a stainless-steel impact mortar under liquid nitrogen and homogenised (20% w/v) in buffer (10 mM Tris, pH 7.4, 1.5 mM EDTA, 10% glycerol, 10 mM monothioglycerol, and 10 mM sodium molybdate) on ice. Centrifugation (50 000 **g**) for 1 h yielded cytosol, which was adjusted with buffer to 2.5 mg protein ml^−1^. All incubations were carried out in triplicate, each containing 100 *μ*l of cytosol, 20 *μ*l of 10 nM 6,7-[^3^H]oestradiol (6,7-[^3^H]E_2_), and 80 *μ*l of antagonist. After incubation overnight at 4°C, tubes received 300 *μ*l of dextran-coated charcoal suspension; tubes were agitated for 15 min, and then centrifuged (1000 **g**) for 15 min. Supernatants were decanted into counting vials, scintillation fluid was added, and protein-bound tritium was determined (Mesfin *et al*, 2001).

### Xenograft assay

Ten million MCF-7 human breast cancer cells were harvested from culture, centrifuged into a pellet, solidified into a fibrin clot, and implanted under the kidney capsule of SCID mice as described previously (Bennett *et al*, 1985, 2002, 2006; Mesfin *et al*, 2001; Parikh *et al*, 2005). Oestrogen supplementation was accomplished by s.c. implantation of a 5 mm silastic tubing capsule containing solid E_2_ inserted on the day of tumour implantation. One milligram of Tam was dissolved in 1 ml 95% ethanol and then diluted to 5 and 0.25 *μ*g ml^−1^ in saline; 0.2 ml of these concentrations was administered to mice. *α*-Fetoprotein-derived peptide was dissolved directly in saline to its appropriate concentrations. Tam was administered p.o. by gavage and AFPep was administered i.p. once a day beginning from 1 day after tumour implantation. The tumour-bearing kidney was exposed during survival laparotomy at 14 and 28 days after implantation and tumour size was measured using a dissecting microscope equipped with an ocular micrometer, noting the long (*D*) and short (*d*) diameters of the tumour. Five replicate mice were included in each treatment group. Mean tumour volume (0.52*d*^2^*D*) was calculated for each group, and used for display of growth curves. Significance of differences between groups was tested using the one-sided Wilcoxon rank sum test.

### Prevention assay

The prevention study utilised methodologies previously described (Grubbs *et al*, 1985; Parikh *et al*, 2005) to test the ability of AFPep, Tam, or the combination of AFPep and Tam to prevent MNU-induced breast cancers in rats. There were 30 rats in each experimental group to assure a 95% probability of detecting a difference between groups (ratios) of 40%, which was the difference seen for pregnancy (Grubbs *et al*, 1983, 1985, 1986; Swanson *et al*, 1997). Power analysis was performed by SOLO software, BMDR Statistical Software Inc. (Los Angeles, CA, USA). Female rats were housed three per cage in a room maintained at 22±1°C, and artificially lighted for 12 h per day. At 50 days of age, rats received a single injection of MNU (50 mg kg^−1^ body weight) or vehicle in the jugular vein. *N*-methyl-*N*-nitrosourea was administered to animals in the various treatment groups according to a predetermined randomisation chart, so as to ensure uniform distribution of the carcinogen across the groups. Beginning 10 days after MNU exposure, treatment with AFPep by s.c. injection, or Tam by oral gavage, occurred once daily. These routes were selected based on prior experience with these drugs in animals (Bennett *et al*, 2002) and potential routes for these drugs in women. To cause minimal stress to the animals, the route control manipulation (i.e., vehicle injection in Tam animals and vehicle gavage in AFPep animals) was not included. The peptide was diluted in saline and 0.2 ml was administered s.c. for 20 days, while Tam was dissolved in corn oil and was administered by oral gavage (0.2 ml) for the same time period. A control group of animals received daily 0.2 ml s.c. injections of saline for the same time period as AFPep administration and experienced the maximal number of tumours. Starting 30 days after MNU treatment, all rats were palpated weekly for detection of mammary tumours, noting number, location, and size. Tumour burden was determined noninvasively using calipers to measure the long (*D*) and short (*d*) diameter of each tumour. Assuming that tumours were ellipsoid shaped, tumour volume was estimated as (0.52*d*^2^*D*). Cage activity of all animals was monitored daily for gross signs of toxicity. At necropsy, body weight and organ weights were assessed as indicators of toxicity.

### Toxicity

To evaluate the effect of AFPep and Tam on uterine hyperplasia, the immature mouse uterine growth assay was utilised (Mesfin *et al*, 2001; Bennett *et al*, 2002; Parikh *et al*, 2005). Briefly, various doses of AFPep and/or Tam were injected i.p. into 2-week old female Swiss pups. Control mice received the vehicle. Twenty-two hours later, uteri were harvested, trimmed free of connective tissue, and weighed. Uterine weights were normalised to donor mouse body weight. There were five replicate mice per group.

In the therapy model (human tumour xenografts growing in mice) and in the prevention model (MNU-induced mammary cancers in rats), the toxicity of AFPep, Tam, and AFP plus Tam was evaluated by monitoring animal body weights, fur textures and cage activity, and weights of specified organs at necropsy.

## RESULTS

**Figure 1:**
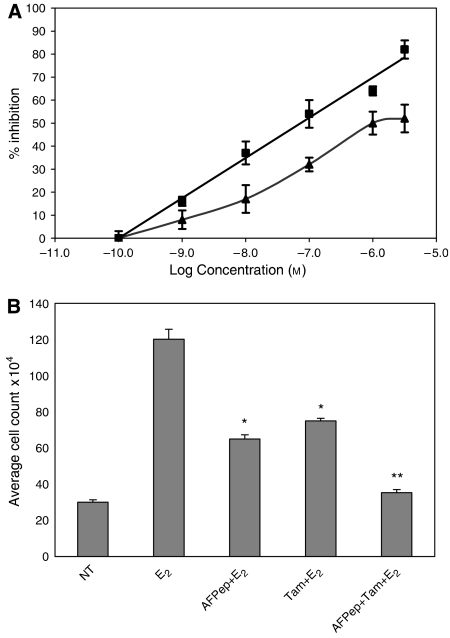
Inhibition of E_2_-stimulated growth of T47D human breast cancer cells by AFPep and Tam. T47D (1 × 10^5^) cells were seeded into wells of collagen-coated plates in oestrogen-free medium. Medium was changed daily. Twenty-four hours after seeding, AFPep and/or Tam were added. One hour later E_2_ (10^−9^ M) was added. Cells were treated daily for 7 days after which cells were harvested and counted in a hemacytometer. Mean viable cell number of six replicate wells ±s.e. was determined. (**A**) Concentration–response curve of each agent alone. Inhibition (%) of E_2_-stimulated growth is reported. (▪) Tam; (▴) AFPep. (**B**) Effect of AFPep and/or Tam on T47D cell proliferation. Mean viable cell number is reported. E_2_, 10^−9^ M; AFPep, 10^−6^ M; Tam, 10^−8^ M (NT, no treatment). ^*^P<0.05 *vs* E_2_ alone, Dunnett's test. ^**^P<0.05 *vs* Tam alone, Scheffe's test.

As shown in Figure 1A, either Tam or AFPep when used alone inhibited the E_2_-stimulated growth of T47D human breast cancer cells in culture. The combination of Tam plus AFPep demonstrated cooperative growth inhibition as exemplified in Figure 1B in which IC_40_ values of Tam plus AFPep produced an 80% inhibition of E_2_-stimulated breast cancer growth. This is especially important when one considers that the IC_40_ of Tam in these experiments was approximately 100-fold lower than the IC_80_ of Tam, suggesting that combination of Tam with AFPep would permit using a lower dose of Tam without overall loss of antitumour activity. Additional results described in subsequent figures support this important concept. In data not shown, it was apparent that, at concentrations ranging from 10^−8^ to 10^−5^ M, neither Tam nor AFPep inhibited the basal (no E_2_) growth of T47D cells, suggesting that their action was directed mainly to the E_2_ stimulation of these cells and was not a nonspecific toxic effect. Also, a control peptide of scrambled sequence did not inhibit the E_2_-stimulated or basal growth of these cells (Bennett *et al*, 2006)

**Figure 2:**
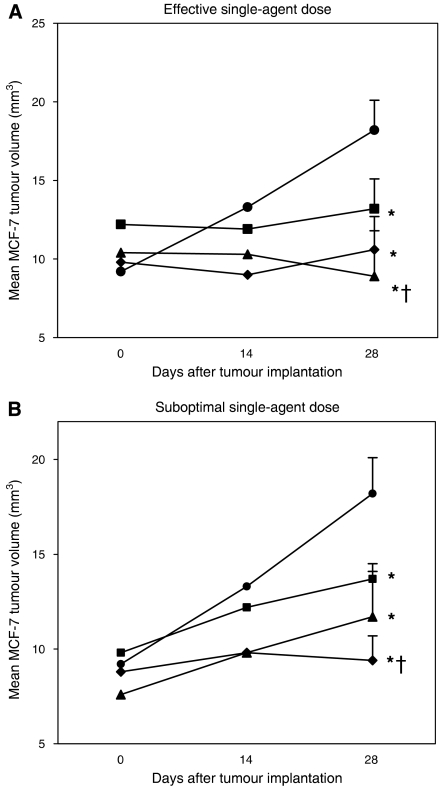
Inhibition of E_2_-stimulated growth of MCF-7 human breast cancer xenografts by AFPep and Tam. Pieces of MCF-7 tumour were implanted under the kidney capsule of SCID mice. Oestradiol was provided via an E_2_-containing silastic tubing subcutaneous implant. Vehicle (•), AFPep (▴), Tam (▪), or AFPep+Tam (⧫) were injected once daily at the doses indicated below beginning from 1 day after tumour implantation. (**A**) Effective single-agent dose (10 *μ*g AFPep and 1 *μ*g Tam). (**B**) Suboptimal single-agent dose (0.1 *μ*g AFPep and 0.05 *μ*g Tam). Mean tumour volume of five replicate mice ±s.e. is reported. ^*^P< 0.05 *vs* vehicle; †*P*<0.05 *vs* Tam alone, Wilcoxon rank-sum test.

Although T47D cells were quite responsive to E_2_ in cell culture, these cells were less reliable when grown as a xenograft in immune-deficient mice, having a take rate (i.e., successful growth) of <60% when implanted in 20 of these mice. In contrast, we have found the MCF-7 human breast cancer cell line to have a tumour take rate of 100% in immune-deficient mice and to be completely dependent on E_2_ for growth in these mice. Hence, MCF-7 xenografts were used as a model of E_2_-dependent human breast cancer being treated with AFPep and Tam *in vivo*. Again, several doses of these drugs were evaluated in preliminary studies in this model to find optimal and suboptimal doses, which were then studied alone and in combination in the studies described herein. As shown in Figure 2A, AFPep at a dose of 10 *μ*g mouse^−1^ day^−1^ or Tam at a dose of 1 *μ*g mouse^−1^ day^−1^, completely prevented the growth of MCF-7 tumour xenografts over a 30-day period. When doses of AFPep or Tam were lowered to 0.1 and 0.05 *μ*g per mouse per day, respectively, tumour growth occurred, although it was less than tumour growth in nontreated mice (Figure 2B). Interestingly, when AFPep and Tam were combined at these suboptimal doses, MCF-7 tumour growth was completely prevented (Figure 2B), again indicating that the maximal effect of Tam can be achieved at a much lower dose of Tam, if it is combined with AFPep. The validation of previous studies (Jacobson *et al*, 1990; Bennett *et al*, 1998, 2002; Mesfin *et al*, 2001; Parikh *et al*, 2005), which have shown that MCF-7 tumours do not grow without E_2_ supplementation is not shown in Figure 2.

**Figure 3:**
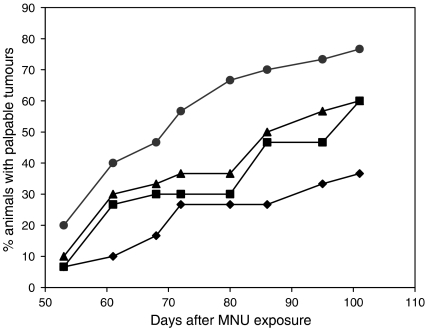
Combination of suboptimal doses of AFPep and Tam prevents breast cancer. Sprague–Dawley female rats (*n*=30 rats group^−1^) received MNU (50 mg kg^−1^) at the age of 50 days. Beginning after 10 days, rats were treated once daily with vehicle (•), or a suboptimal dose of Tam (▪, 50 ng animal^−1^, p.o.), or with a suboptimal dose of AFPep (▴, 100 *μ*g animal^−1^, s.c.) once daily for 20 days, or with both (⧫). Animals were palpated weekly. The incidence of tumours is shown as a function of time after carcinogen treatment.

As a model for preventing the development of breast cancer, we utilised the well-characterised system of carcinogen (MNU)-induced mammary cancer in Sprague–Dawley rats (Grubbs *et al*, 1983, 1985, 1986; Swanson *et al*, 1997). When carcinogen-exposed rats were treated with Tam at doses of 6.25 *μ*g animal^−1^ day^−1^ or higher, or AFPep at 270 *μ*g animal^−1^ day^−1^, there was significant inhibition of tumour formation, as shown in Table 1. These results are similar to those of Moon *et al* (1992, 1994) using Tam and to Parikh *et al* (2005) using AFPep. At a suboptimal dose of Tam or of AFPep, inhibition of tumour formation was not significantly diminished compared to controls (Table 1, Figure 3). When these two drugs were used in combination at suboptimal doses, their combined chemopreventive contribution resulted in a decrease in tumour incidence (Figure 3, Table 1), which was significantly below that seen in control (*P*=0.04, Fisher's exact). Doses of AFPep (100 *μ*g animal^−1^ day^−1^;) or of Tam (0.05 *μ*g animal^−1^ day^−1^) given to the rats were held constant during the 20-day treatment interval. During this interval, all animals in all groups gained weight from 165 to 190 g, indicating that treatments did not affect body weight. Table 1 shows that latency increased significantly, multiplicity decreased, and tumour burden (volume) decreased significantly following combination treatment with the lower doses of AFPep in combination with Tam.

In all previous work with high doses of AFPep, no evidence of toxicity or side effects has been detected (Mesfin *et al*, 2001; Bennett *et al*, 2002, 2006; Parikh *et al*, 2005). In this study in which AFPep or combination of AFPep and Tam were administered to rats at therapeutic doses for 2–3 weeks, there was no effect on body weight, fur texture, or cage activity during the lifespan of the animals, or on organ weights obtained at necropsy (Table 1). At these low doses, Tam had no effect on uterine growth, whether or not AFPep was present.

**Figure 4:**
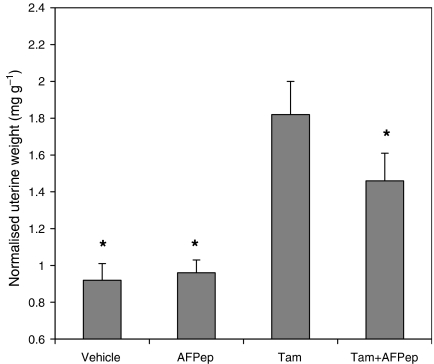
Effect of AFPep and Tam on growth of immature mouse uterus. Mice were injected i.p. with Tam (1 *μ*g), AFPep (10 *μ*g), or AFPep plus Tam at the doses already indicated. In the case of the combination, AFPep was given 1 h before Tam. Twenty-two hours after treatment, uteri were harvested, weighed, and normalised to mouse body weight. Mean uterine weight of five replicate mice per group ±s.e. is reported. ^*^P<0.05 *vs* Tam alone, Dunnett's test.

A side effect of Tam is the induction of uterine hyperplasia in approximately 30% of the patients taking this drug, and this progresses to uterine cancer in roughly 0.2% of users (Fisher *et al*, 1994; Assikis and Jordan, 1995). As shown in Figure 4, Tam stimulates the growth of immature mouse uterus. *α*-Fetoprotein-derived peptide does not stimulate uterine growth. Moreover, AFPep significantly inhibits the Tam-induced growth of the immature mouse uterus. Therefore, in women, adding AFPep to Tam would be expected to diminish the uterine growth side effect of Tam.

**Figure 5:**
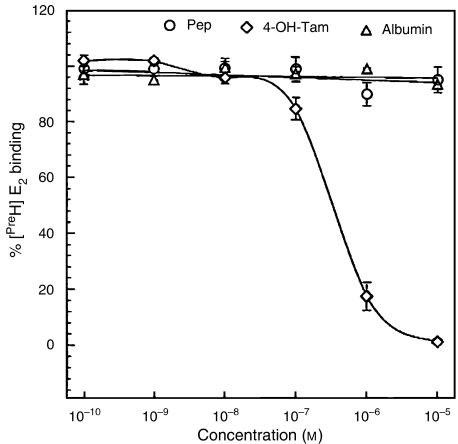
Effect of AFPep and Tam on binding of E_2_ to the ER. Rabbit uterine cytosol was used as a source of ER. Test agents (80 *μ*l) at the final concentrations indicated were incubated in triplicate with 100 *μ*l of cytosol, and 20 *μ*l of 10 nM 6,7-[^3^H]E_2_ (50 Ci mmol^−1^). The concentration of the [^3^H]E_2_ complex with receptor in the presence of the test agent is expressed as a percentage of the amount of complex formed in the absence of test agent.

Mechanistically, AFPep is quite different from Tam. As shown in Figure 5, AFPep did not inhibit the binding of [^3^H]E_2_ to ER, while Tam demonstrated the concentration-dependent inhibition of E_2_ binding expected from an ER antagonist. Also, in other studies, we have shown that AFPep did not stimulate phosphorylation of ER at serine 118 (Bennett *et al*, 2006), which is the phosphorylation site stimulated by E_2_ (Kato *et al*, 1995). In contrast, it has been shown that Tam stimulates phosphorylation of ER at serine 118, following its binding to this receptor (Chen *et al*, 2002). Thus, Tam and AFPep impact the ER in quite different ways, and yet work cooperatively together to inhibit oestrogen-dependent breast cancer growth.

## DISCUSSION

Tamoxifen has been a very effective drug for the treatment of ER+ breast cancer (Lippman and Bolan, 1975; Early Breast Cancer Trialists' Collaborative Group, 1992; Fisher *et al*, 1998; Jordan, 1999a). However, its effectiveness wanes with time, and after 2–5 years of treatment, most ER+ tumours become refractory to Tam (Early Breast Cancer Trialists' Collaborative Group, 1992; Jordan, 1999a). Also, there are some ER+ breast cancers that are unresponsive to Tam at first presentation (Jensen and DeSombre, 1996), and there are others that, after chronic treatment with Tam, actually become growth-stimulated by Tam (Canney *et al*, 1987; Gottardis and Jordan, 1988; Howell *et al*, 1992). There are no good tests to differentiate between these phenotypes, and it is possible that seeds of each phenotype are present when breast cancer is first diagnosed. One logical approach to this problem is to treat with combinations of anti-endocrine agents using the prescribed combination principles of each agent being active alone, having different mechanisms of action and having non-cross-reacting host toxicities. With this strategy, those phenotypes not held in check by Tam would theoretically be arrested by the agent(s) combined with Tam. Zaccheo *et al* (1991, 1993) have validated this principle by showing that Tam plus examestane, an aromatase inhibitor, was more effective than Tam alone in stopping breast cancer growth, and now combinations of Tam plus aromatase inhibitors are showing promise clinically (Abrial *et al*, 2006).

The data in the study reported herein support three independent concepts: AFPep has antitumour activity on its own, it adds to the antitumour activity of Tam through a mechanism distinct from Tam, and it can reduce the toxicity of Tam by decreasing the uterine hyperplasia of Tam and by allowing dose reduction of Tam without loss of antitumour activity.

We have been studying AFPep as an inhibitor of oestrogen-dependent growth (Mesfin *et al*, 2001; Bennett *et al*, 2002; Parikh *et al*, 2005). *α*-Fetoprotein-derived peptide is not an ER antagonist, nor an ER partial agonist, making its mechanism different from Tam (Parikh *et al*, 2005; Bennett *et al*, 2006). Its function, at least in part, is to inhibit the phosphorylation of ER that follows ligand binding to ER. Phosphorylation has been shown to be necessary for full functionality of ER (Kato *et al*, 1995). Furthermore, AFPep does not share the toxicities of Tam. In fact, in this study and in studies reported elsewhere (Bennett *et al*, 2002), AFPep interferes with the uterine hyperplasia induced by Tam (i.e., reduces the toxicity of Tam). The fact that AFPep inhibits Tam stimulation of growth in the uterus suggests that AFPep may inhibit not only that toxicity of Tam in humans but also those breast cancer phenotypes that are actually stimulated by Tam, as well as those that are indifferent to Tam. *α*-Fetoprotein-derived peptide clearly fits the combination principles of being active when used alone, having a different mechanism of action from Tam, and having non-cross-reacting host toxicity with Tam. Hence, it was eminently logical to postulate that AFPep would be beneficial in combination with Tam, and the results of this study provide the data to support this contention. Not only did AFPep plus Tam inhibit the growth of an extant oestrogen-dependent human breast cancer better than Tam alone, but also the combination was more effective than Tam alone in preventing carcinogen-induced mammary cancers in rats. It should not go unnoticed that in the effective combination, the dose of Tam was substantially lower than that employed for Tam alone, suggesting that such dose reduction may, in itself, alleviate Tam's toxicities (Jordan, 1999b). In addition, the data in Figure 4 suggest that AFPep will further alleviate some of those toxicities as it inhibits Tam-induced uterine hyperplasia. No toxicities from AFPep have become evident. Since it is derived from a natural human fetal protein (*α*-fetoprotein, AFP), and since its active dose is below the fetal physiological level of AFP, it is possible that the side effects associated with AFPep will, at most, be minimal. Thus, it is highly unlikely that it will add to any of the toxicities of Tam, and, as mentioned above, should alleviate some of those toxicities while contributing to the antitumour activity of Tam. While the study reported herein was ongoing, it was found that AFPep was active by the oral route (Bennett *et al*, 2006). Hence, the discomfort of chronic injections will not be an issue with use of AFPep as a therapeutic or preventive agent.

Using a variety of models and species (human breast cancer cells growing in culture, human breast cancer cells growing in a an immune-deficient mouse model of therapy, or chemically induced mammary carcinoma in a rat prevention model), the results of this study have shown that AFPep works well in combination with Tam for the treatment and prevention of experimental breast cancer. Its mechanism is quite different from that of Tam, and its toxicity is minimal. As such, AFPep warrants further development as a new agent that could be used in combination with Tam, or perhaps even used as a stand-alone agent, for the treatment or prevention of breast cancer in humans.

## Figures and Tables

**Table 1:** Effect of AFPep and Tam on prevention of breast cancer in MNU-exposed rats

**Treatment**	**Dose (*μ*g/rat/day)**	**Incidence,^a^ % (*P*-value^b^)**	**Multiplicity^c^ (tumours/rat)**	**Latency^d^ (days)**	**Volume^e^ (cm^3^)**	**Body weight (g)**	**Uterine weight (g)**	**Heart weight (g)**
None (control)	—	78	2.1	80±31	69.0	283±30	0.54±0.15	1.10±0.14
AFPep	270	40 (0.02)	0.5	88±16	34.5^*^	280±29	0.62±0.17	1.09±0.13
AFPep	100	63 (0.16)	1.1	97±33	39.1^*^	285±24	0.52±0.15	1.12±0.14
Tam	6.25	26 (0.001)	0.5	85±15	70.2	273±28	NA	NA
Tam	0.05	73 (0.21)	1.3	95±36	62.4	282±31	0.49±0.17	1.12±0.13
AFPep+Tam	100+0.05	52 (0.04)	0.9	110±33	32.6^*^	282±29	0.51±0.12	1.09±0.15

AFPep=*α*-fetoprotein-derived peptide; MNU=*N*-methyl-*N*-nitrosourea; NA=not applicable; Tam=tamoxifen.

^*^P<0.05 *vs* control.

a Percent of rats with one or more tumours when killed (approximately 120 days after MNU exposure).

b *P*-value (calculated according to Fisher's exact test) compared to control group.

c Multiplicity is defined as total number of tumours/number of rats.

d Mean number of tumour-free days±s.d.

e Sum of the volumes of tumours in each group.
